# Impact of multiple environmental factors on influenza–like illness in Fujian Province, China, 2015–2023: a multicity study

**DOI:** 10.3389/fpubh.2025.1656880

**Published:** 2025-09-04

**Authors:** Xiaoyan Yao, Yanhua Zhang, Yuemei Hu, Enjun Cui, Fen Lin, Guangmin Chen, Jianfeng Xie, Yuwei Weng, Kuicheng Zheng

**Affiliations:** ^1^The School of Public Health, Fujian Medical University, Fuzhou, Fujian, China; ^2^Fujian Provincial Center for Disease Control and Prevention, Fujian Provincial Key Laboratory of Zoonosis Research, Fuzhou, China; ^3^Department of Pharmacy, Weihai Municipal Hospital, Weihai, China

**Keywords:** influenza-like illness, environmental epidemiology, distributed lag non-linear model, multi-city study, air pollution, temperature variability

## Abstract

**Introduction:**

Influenza-like illness (ILI) represents a significant global public health challenge influenced by environmental factors. While previous studies have demonstrated associations, most have been limited to single-city analyses with inconsistent findings. This multi-city study systematically examines the effects of meteorological and air pollution factors on ILI across diverse urban environments.

**Study design:**

We analyzed daily ILI surveillance data (2015–2023) from 18 sentinel hospitals across nine Fujian Province cities, combined with concurrent air quality and meteorological data. Using LASSO regression for variable selection, we employed distributed lag non-linear models (DLNMs) to characterize exposure-response relationships in each city, followed by random-effects multivariate meta-analysis to pool estimates and assess heterogeneity.

**Results:**

A total of 2,995,909 ILI cases were collected. ILI cases in nine cities of Fujian Province exhibited significant seasonal fluctuations, peaking in winter or early summer. LASSO regression identified temperature, NO₂, and SO₂ as key environmental factors. Our results indicated that the combined cumulative effect of average temperature on ILI across nine cities decreased with rising temperatures, with a risk peak at −0.1°C. The NO₂–ILI association was non–linear, resembling an inverted “U” shape, with a risk peak at 40.5 μg/m^3^. SO₂ exposure had a large degree of heterogeneity in its effect on ILI.

**Conclusion:**

This study provides robust evidence that ambient temperature and NO₂ levels significantly influence ILI risk in Fujian Province, with distinct exposure-response patterns. Public health strategies should prioritize cold-weather preparedness and air quality management, particularly during high-risk seasons. Our two-stage analytical approach addresses previous limitations in multi-city environmental health studies.

## 1 Introduction

Influenza-like illness (ILI) is a clinical syndrome characterized by symptoms such as fever and other flu-like manifestations (1). Serving as a crucial non-specific indicator in influenza surveillance, ILI not only reflects the epidemic trends of influenza but also provides an effective approach for investigating its influencing factors (2). According to data from the World Health Organization (WHO), influenza continues to circulate globally, with an annual incidence rate of 5–10% in adults and as high as 20–30% in children. It results in 3 to 5 million severe cases and 290,000 to 650,000 respiratory deaths annually worldwide (3). In China, influenza is classified as a Category C notifiable infectious disease, with an estimated annual average of approximately 88,100 influenza-associated excess deaths (4). Given its high transmissibility and potential to cause severe outcomes, the disease burden attributed to influenza is substantial. Therefore, a systematic evaluation of the environmental drivers influencing influenza virus transmission is of significant importance for informing and improving public health prevention and control strategies. The epidemic of ILI exhibits significant seasonal characteristics. According to the influenza surveillance reports released by the Chinese Center for Disease Control and Prevention, ILI peaks in northern China are primarily concentrated in the winter and spring, while southern China experiences peaks both in winter–spring and summer (5). This seasonal pattern may be influenced by various factors, including the host’s immune status, socio–economic conditions, and environmental factors.

Although the association between environmental factors and the transmission of ILI has become a research focus, substantial academic controversy remains, particularly regarding the effects of temperature and humidity, for which significant inconsistencies have been observed across studies. As a key meteorological variable influencing influenza spread, the relationship between temperature and ILI risk exhibits notable regional heterogeneity. An analysis by Cao et al. across 122 countries and regions revealed an inverted U-shaped curve for the cumulative relative risk of influenza in relation to temperature, though the peak risk temperature varied geographically: influenza risk peaked between 6 and 14° C in Africa, was highest below 3° C in Asia, and showed distinct epidemic peaks above 11° C in the Americas and Oceania (6). In contrast, a study by Yang et al. conducted in the United States reported an inverse correlation between temperature and ILI risk, indicating that lower temperatures were associated with higher ILI incidence (7). This finding is consistent with epidemiological observations in northern China, where the seasonal influenza peak in winter and spring coincides with periods of lower temperatures.

The relationship between humidity and influenza transmission also demonstrates complex patterns. Research by Li et al. in Hong Kong indicated a significant negative correlation between absolute humidity and influenza incidence, suggesting that higher humidity levels may suppress viral spread (8). However, emerging evidence points to a U-shaped association, wherein risk of influenza transmission is elevated under both low and high absolute humidity conditions (9). The heterogeneity in these findings may stem from methodological variations, region-specific factors, and interactions among environmental variables. Therefore, there is an urgent need for more systematic and integrated research frameworks to elucidate the underlying mechanisms through which environmental factors influence influenza transmission dynamics.

The impact of air pollutants on influenza transmission remains a subject of inconsistent and even contradictory findings across existing studies. Multiple studies conducted in China have reported positive correlations between pollutants—including particulate matter (PM), sulfur dioxide (SO₂), nitrogen dioxide (NO₂), ozone (O₃), and carbon monoxide (CO)—and the incidence of ILI (10). However, these associations exhibit notable spatiotemporal heterogeneity, with more pronounced effects observed during cold seasons, in eastern and central regions, as well as in humid and densely populated provinces. For instance, research from Beijing indicated that elevated PM₂.₅ concentrations during influenza seasons were significantly associated with increased ILI visits (11). Similarly, a study in Jinan demonstrated that PM₂.₅, PM₁₀, CO, and SO₂ elevated the risk of ILI (12). In contrast, a report from Hefei suggested a negative correlation between PM₁₀ and clinical ILI incidence, while a study conducted in Nanjing found no significant association between PM₂.₅, PM₁₀, or NO₂ and ILI risk among individuals aged 25 and above (13, 14). These inconsistent results underscore the potentially strong temporal and spatial dependence of the effects of air pollutants on influenza transmission, highlighting the need for further systematic investigation into the underlying mechanisms.

Fujian Province presents a particularly compelling case study due to its unique subtropical monsoon climate and rapid urbanization. The region experiences high humidity, frequent temperature fluctuations, and complex air pollution profiles – all potential modifiers of ILI transmission dynamics (15). However, current understanding remains limited by single-city study designs that fail to capture regional heterogeneity or adequately address the non-linear, delayed effects of environmental exposures. This study addresses these gaps through a comprehensive multi-city analysis across nine urban centers in Fujian, employing distributed lag non-linear modeling to simultaneously evaluate meteorological and pollution effects while accounting for spatial variability and temporal delays, with the ultimate goal of providing robust evidence for region-specific ILI prevention strategies and advancing methodological approaches for environmental health research in subtropical climates.

## 2 Methods

### 2.1 Data sources

The daily numbers of reported ILI cases reported from 1 January 2015 to 31 December 2023 were obtained from the National Influenza Center of China (CNIC). The dataset includes ILI surveillance data from 18 sentinel hospitals across nine cities in Fujian Province (Fuzhou, Longyan, Nanping, Ningde, Putian, Quanzhou, Sanming, Xiamen, and Zhangzhou), with 2 hospitals in each city. The surveillance content covers both epidemiological and etiological monitoring, with long–term continuous monitoring of ILI cases in outpatient and emergency departments, as well as the dynamic changes in the percentage of ILI cases among outpatient visits at sentinel hospitals. The definition ILI used in this study strictly follows the criteria outlined in the China National Influenza Surveillance Technical Guide (2017 Edition), which is consistent with the influenza surveillance case definition recommended by the WHO (16). Specifically, it is defined as the outpatients who had acute respiratory infection with body temperature more than 38°C and either cough or sore throat.

We also collected meteorological and air pollutants data from the nine cities of Fujian Province for the period from January from 1 January 2015 to 31 December 2023. Air pollutants data was obtained from the China Air Quality Online Monitoring and Analysis Platform^1^, which includes daily average concentrations of AQI, PM2.5 (μg/m^3^), PM10 (μg/m^3^), SO₂ (μg/m^3^), NO₂ (μg/m^3^), and O₃ (μg/m^3^). Meteorological data was sourced from the China Meteorological Data Service Center^2^, including daily average temperature (°C), wind speed (m/s), and daily accumulated rainfall (mm).

### 2.2 Statistical analysis

#### 2.2.1 LASSO regression

We first performed descriptive analysis of the meteorological and air pollutants data as well as the ILI from the nine cities in Fujian Province. By plotting the time trend of ILI case numbers, we observed its annual variation pattern. Furthermore, the normalized seasonal distribution was obtained by dividing the monthly case counts by the total number of cases in that year, and the peak epidemic period was identified by comparing the monthly averages.

LASSO regression was used to control for multicollinearity among variables and to filter variables (17). We initially fitted a generalized linear model using penalized maximum likelihood. Then, the cross–validation method was used to calculate the parameter *λ*, which minimizes the mean cross–validation error. Finally, the parameter λ was reintroduced into the equation to calculate the optimal coefficients for each variable, and the selected variables were used for subsequent analysis (18).

#### 2.2.2 DLNM model

In the first stage, for the total population of Fujian Province, ILI cases are considered rare events. Additionally, in numerous epidemiological studies both domestically and internationally, it is often assumed that the number of deaths, outpatient visits, or hospitalizations approximately follows a Poisson distribution (19, 20). Therefore, this study assumes that the number of ILI visits follows a Poisson distribution. Given the characteristics of the data in this study, we used a Generalized Additive Model (GAM) with a log link and allowed Poisson autocorrelation to establish the logarithm of the expected ILI cases in Fujian Province and the relationships between environmental factors, combined with a Distributed Lag Non–linear Model (DLNM) to quantify the single and cumulative effects of environmental data on daily ILI cases in each city of Fujian. Considering the incubation period (3–5 days) and infectious period (approximately 2 weeks) of most respiratory viral infections (21), the lag range was set to 0–14 days to fully cover the lag effects. A cross–basis function was chosen, applying natural cubic splines to both the independent variables and their lags to display the exposure–lag–response associations. Natural cubic splines with 7 degrees of freedom (df) were used for the time variable to suppress long–term trends. The model also adjusted for the effects of the day of the week (DOW). The final basic form of the model is as follows:

Ln [E (Yt)] = β_1_(NO2)t + β_2_(SO2)t + β_3_(Tem)t + ns (Time, df) + Dow.

In the equation, Yt represents the number of ILI reports in a prefecture–level city on day t; β_1_, β_2_,β_3_ are the regression coefficient; t is the value of a specific environmental factor on day t to be studied; ns() is the natural spline function used to adjust for the non-linear effects of variables; Time is the time variable (a sequence of study days: 1, 2, 3, 4, …, N); df is the degrees of freedom; Dow is the weekday dummy variable, used to control for confounding factors related to periodic healthcare-seeking behavior (22). A natural cubic spline function with 7 dfs/year was used to eliminate the long–term and seasonal trends in the ILI visit counts.

This study used the median values of each exposure as the baseline evaluation level. First, we estimated the relative risk of daily ILI under the mean conditions of environmental factors and obtained both the single lag effects and cumulative effects. The mean values of environmental factors represent the most common environmental quality conditions in the cities of Fujian Province. Next, the P5 and P95 values of the exposure levels were used as cutoff points for low and high exposure levels, respectively, to estimate the impact of extreme exposure conditions on ILI. Predictions were made using the crosspred function, and the original response curves, 3D plots, and contour plots were generated to visually display the overall exposure–lag–response relationship, assessing the effects of each exposure factor on ILI and their lag effects.

In the second stage, the exposure–response relationships for the nine cities obtained in the first stage were simplified into simpler one–dimensional coefficients of exposure and lag dimensions, along with the corresponding covariance matrices. These were then aggregated using a multivariate meta–analysis and restricted maximum likelihood estimation (REML) (23). For each air pollutant and meteorological factor, the lag response relationships at specific exposure levels (including the 5th and 95th percentiles) were extracted. Additionally, the lag effects of each air pollutant level were summed to represent the cumulative relationship between air pollutants and ILI. At the regional level, a multivariate regression intercept model was used for meta–regression analysis of the dimension–reduced city–specific effect estimates, yielding region–specific merged lag–response relationships and cumulative exposure–response relationships.

The residual heterogeneity was tested using the multivariate extension of the Cochran *Q* test and the *I*^2^ statistic (residual heterogeneity was considered statistically significant when *p* < 0.05) (24). In this study, the exposure–response degree was quantified using the relative risk (RR). We quantified the uncertainty of the point estimates in the study by calculating the RR and the 95% confidence interval (95% CI) of RR.

#### 2.2.3 Sensitivity analysis

We evaluated the robustness of our model through sensitivity analyses in the following three aspects: First, we altered the degrees of freedom (df) for the time trend from 6 to 8 (original df = 7) for the temporal trend and refitted a three-predictor exposure-response model including temperature, NO₂, and SO₂ to examine the influence of temporal control strategies on the estimates of the main effects. Second, we excluded two cities with extreme population sizes—Fuzhou (the highest population) and Sanming (the lowest population)—to assess the impact of demographic outliers on the overall results. Third, we incorporated additional environmental variables, including other pollutants and meteorological factors, as covariates in the model to evaluate potential confounding effects on the estimates of the primary exposure variables. Through these multidimensional assessments, we ensured the stability and reliability of our findings under varying model specifications and data structures.

All analyses were performed using R software (version 4.0.3), and the R packages “dlnm” and “mvmeta” were used to fit the DLNM and multivariate meta–regression models, respectively. A two–sided *p* < 0.05 was considered statistically significant for all statistical tests.

## 3 Results

### 3.1 Descriptive analysis

**Figure 1:**
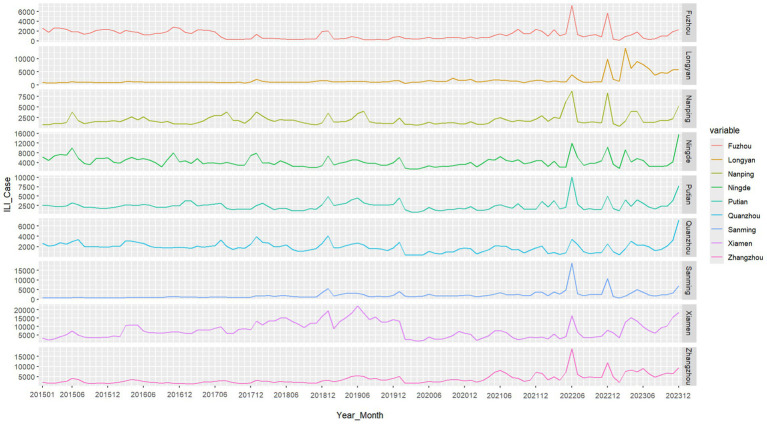
Time series of ILI cases in 9 cities in Fujian Province between 2015 and 2023. Generated based on the monthly ILI consultation rates from various prefecture-level cities in Fujian Province.

**Figure 2:**
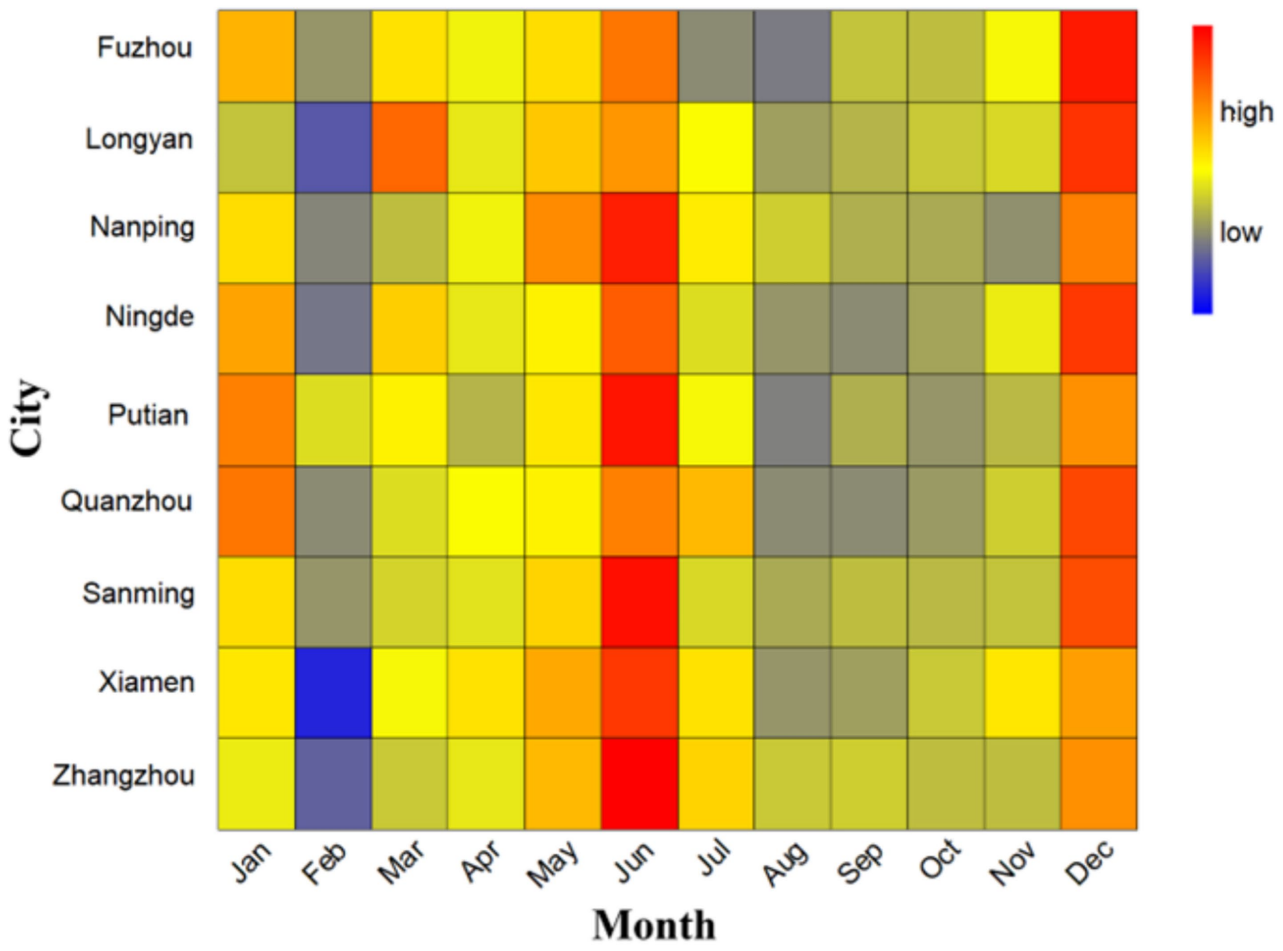
Seasonal distribution of ILI cases in 9 cities in Fujian Province, 2015–2023. The term “high” represent values with above-average ILI activity, indicating a greater concentration or frequency of cases relative to the average seasonal distribution. The term “low” typically represent values with below-average ILI activity, indicating a lower concentration or frequency of cases relative to the average.

From January 1, 2015, to December 31, 2023, a total of 2,995,909 ILI cases were collected in Fujian Province (Supplementary Table S1). The annual ILI cases peaked in 2023 (475,540 cases, 15.87%) and reached its nadir in 2020 (209,769 cases, 7.00%). Time trend line chart revealed consistent seasonality across nine cities (Figure 1), with dual annual peaks occurring in winter (December–February) and early summer (May–June), as confirmed by normalized seasonal distribution patterns (Figure 2). The pollutant values for each city are shown in Supplementary Table S2. The time series data of the four air pollutants (NO₂, PM₁₀, PM₂.₅, and AQI) in the nine cities all show similar periodicity and relative stability (Supplementary Figure S1).

### 3.2 LASSO regression result

LASSO regression analysis was used to determine the selection of covariates that might overfit the model due to multicollinearity. The optimal *λ* value was 4.49 (one standard error below the minimum standard), with three non-zero coefficients represented in sparse matrix format. This study evaluated multiple climate variables and air pollutants. Finally, the variables of average temperature, SO₂, and NO₂ were included in the analysis to avoid multicollinearity, as shown in Table 1.

**Table 1:** Sparse matrix of covariates coefficients.

Factors	Coefficients
Intercept	93.1886548
Air Quality Index (AQI)	NE
PM10 (μg/m^3^)	NE
PM2.5 (μg/m^3^)	NE
Mean temperature (°C)	0.4152534
SO₂ (μg/m^3^)	−2.3726399
NO₂ (μg/m^3^)	0.8637550
O_3_ (μg/m^3^)	NE
Precipitation (mm)	NE
Wind speed (m/s)	NE

NE, not estimated; PM2.5, particulate matter of <2.5 μm; PM10, particulate matter of <10 μm; AQI, Air Quality Index; SO₂, sulfur dioxide; NO₂, nitrogen dioxide; O_3_, ozone.

### 3.3 The relationship between NO₂, SO₂, temperature, and ILI in nine cities

**Figure 3:**
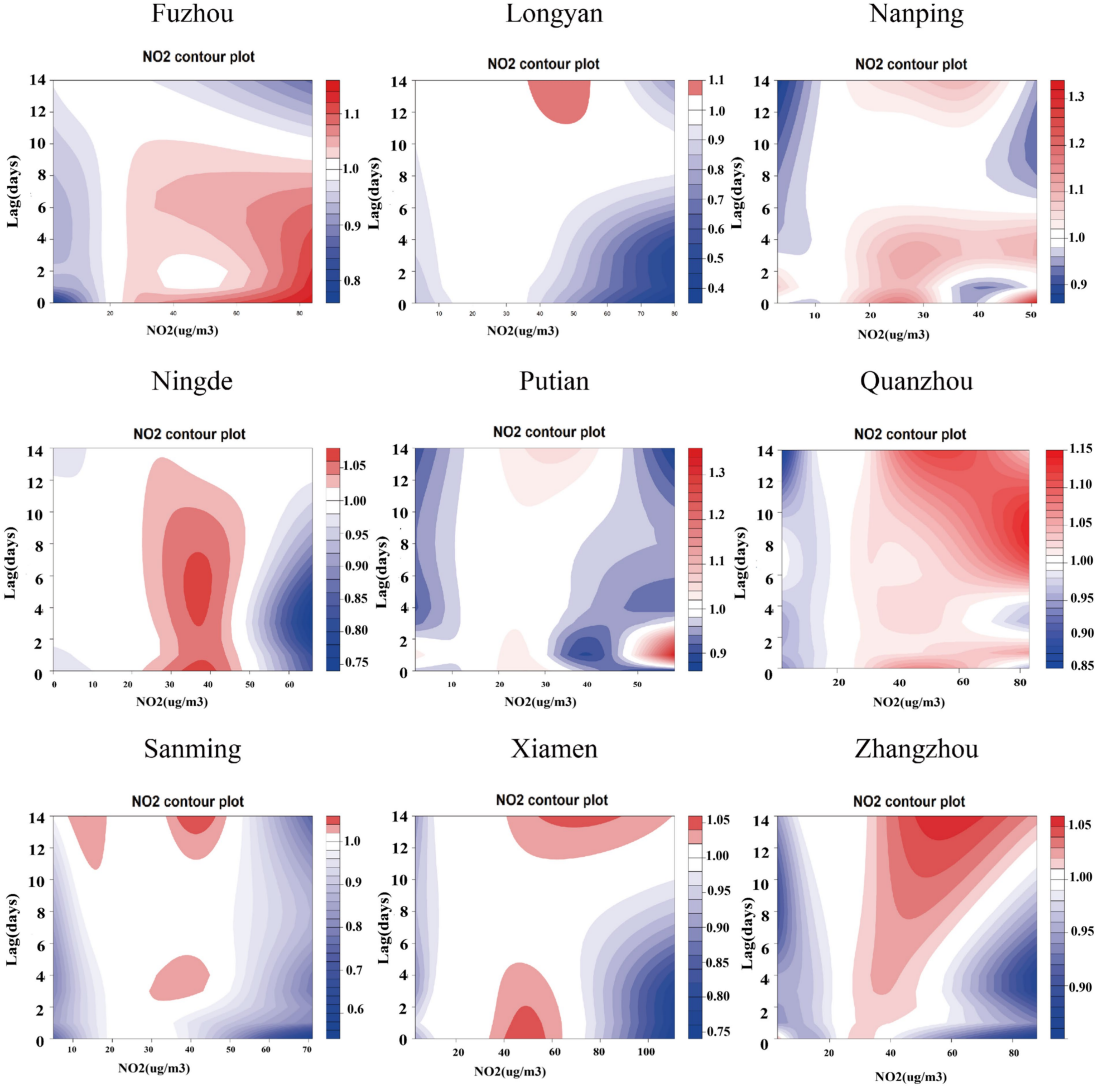
Contour plots of the relative risks of NO₂ on ILI cases in 9 cities in Fujian Province from 2015 to 2023. The reference level was set to the median value of the corresponding variable. The *Y*–axis represents the lag period from 0 to 14 days. The *X*–axis represents the range of observations for each variable. RR stands for relative risk, red stands for RR > 1, white stands for RR = 1, and blue stands for RR < 1.

**Figure 4:**
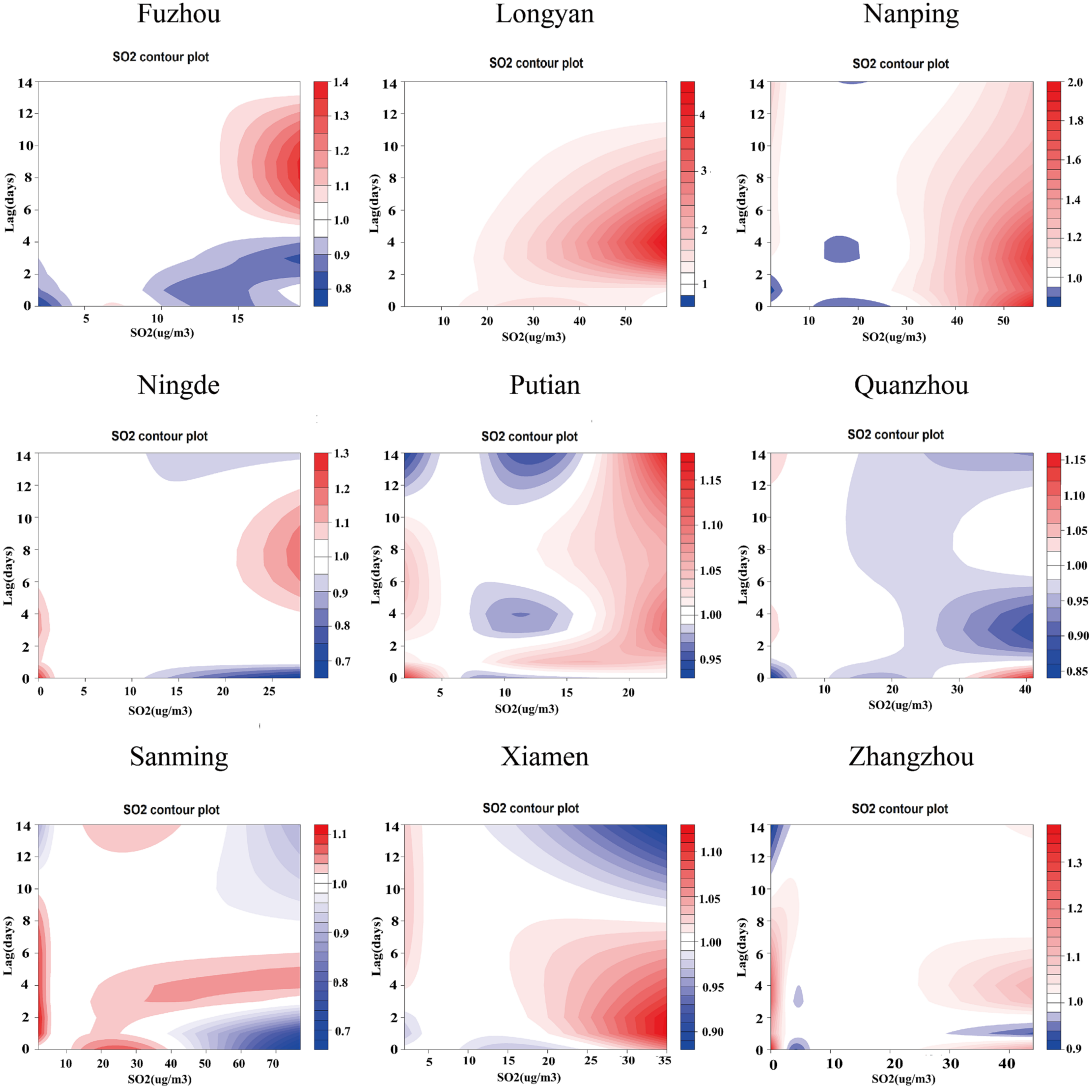
Contour plots of the relative risks of SO₂ on ILI cases in 9 cities in Fujian Province from 2015 to 2023. The reference level was set to the median value of the corresponding variable. The *Y*–axis represents the lag period from 0 to 14 days. The *X*–axis represents the range of observations for each variable. RR stands for relative risk, red stands for RR > 1, white stands for RR = 1, and blue stands for RR < 1.

**Figure 5:**
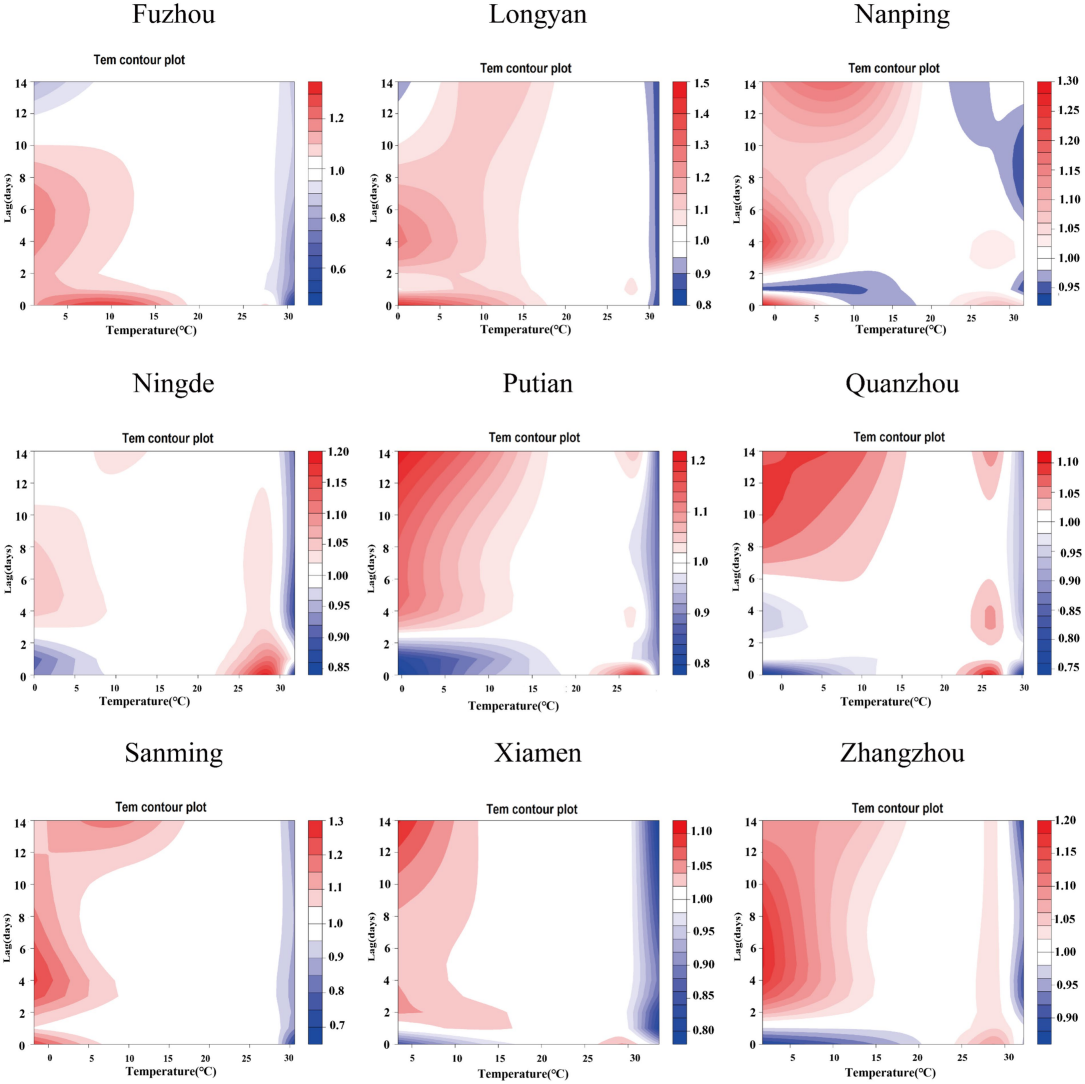
Contour plots of the relative risks of temperature on ILI cases in 9 cities in Fujian Province from 2015 to 2023. The reference level was set to the median value of the corresponding variable. The *Y*–axis represents the lag period from 0 to 14 days. The *X*–axis represents the range of observations for each variable. RR stands for relative risk, red stands for RR > 1, white stands for RR = 1, and blue stands for RR < 1.

Supplementary Tables S3, S6 present comprehensive single-lag and cumulative effect estimates, while three-dimensional (Supplementary Figures S2, S5, S8) and contour plots (Figures 3–5) visualize exposure-lag-response relationships.

For NO₂, significant spatial heterogeneity emerged: Fuzhou, Nanping, and Ningde showed acute effects (0–6 day lags) at 30–60 μg/m^3^, whereas Xiamen exhibited a unique bimodal pattern – initial risk elevation at high concentrations/short lags transitioning to protective effects (RR < 1) at ~5-day lags. Low NO₂ exposure (5th percentile) conferred protection across all cities, most markedly in Fuzhou, decreasing from RR = 0.875 (95% CI: 0.854–0.896) at lag 0 to 0.491 (95% CI: 0.471–0.512) at lag 14. Conversely, high exposure (95th percentile) increased risk cumulatively (peak at lag 14).

SO₂ effects demonstrated similar regional variation, with Fuzhou showing maximal risk at 19 μg/m^3^ with 9-day lag (RR = 1.364; 95%CI: 1.268–1.467) versus Xiamen’s negative associations. Temperature effects followed a consistent thermal gradient: lower temperatures (10–20°C) with longer lags increased risk (RR > 1), while higher temperatures (>30°C) with shorter lags were protective (RR < 1). Cumulative 14-day effects revealed cold-temperature vulnerability in all cities except Ningde and Quanzhou, with 5th percentile exposures consistently elevating risk (single-day RR > 1).

### 3.4 Pooled effects of NO₂, SO₂, temperature on ILI

**Figure 6:**
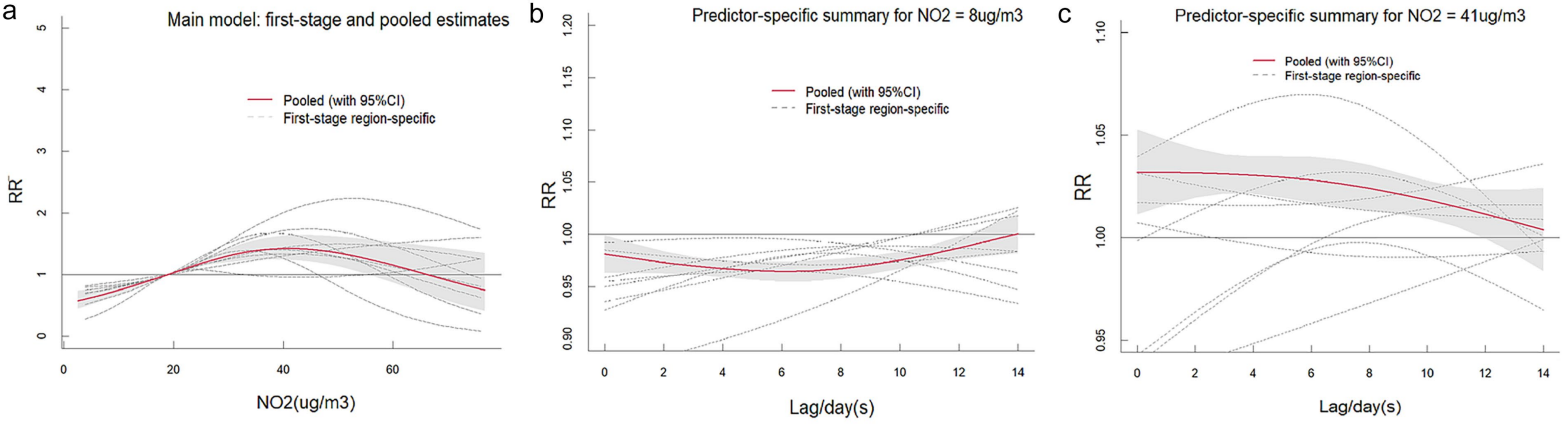
The pooled effects of NO₂ on ILI in Fujian, 2015–2023. The picture **(a)** shows the overall cumulative effects over lag 0–14 days in 9 cities, the two pictures describe **(b,c)** the pooled effects at predictor–specific (95th and 5th percentile of NO₂). The dotted lines represent the different effects of 9 cities, the red line represents the pooled effect and the shaded area is the confidence interval (CI with 95%). The reference level was set to the median value of the corresponding variable.

**Figure 7:**
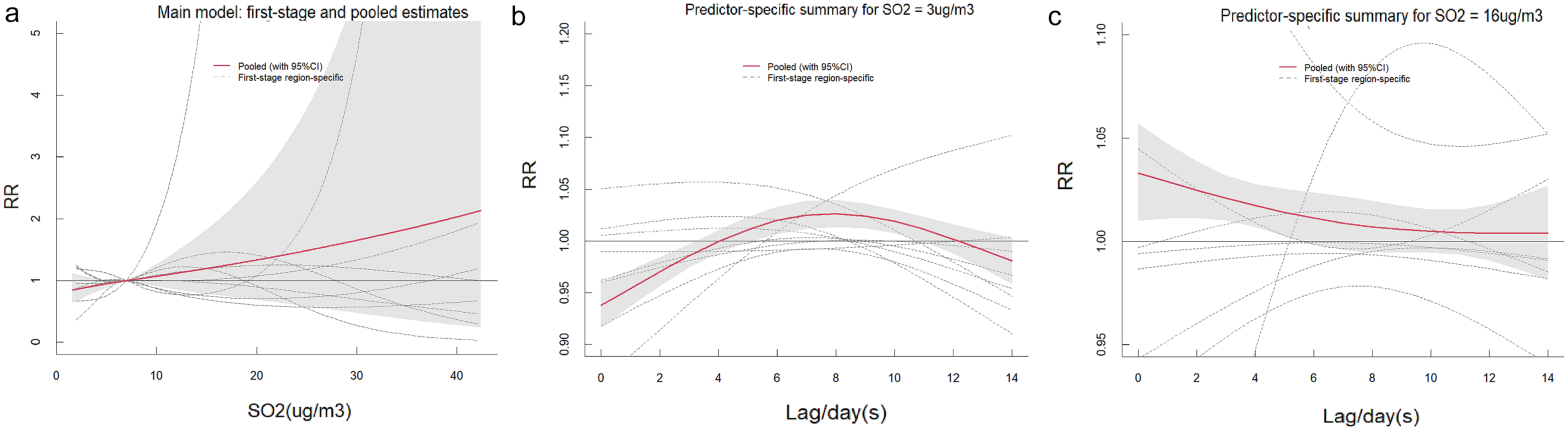
The pooled effects of SO₂ on ILI in Fujian, 2015–2023. The picture **(a)** shows the overall cumulative effects over lag 0–14 days in 9 cities, the two pictures describe **(b,c)** the pooled effects at predictor–specific (95th and 5th percentile of SO₂). The dotted lines represent the different effects of 9 cities, the red line represents the pooled effect and the shaded area is the confidence interval (CI with 95%). The reference level was set to the median value of the corresponding variable.

**Figure 8:**
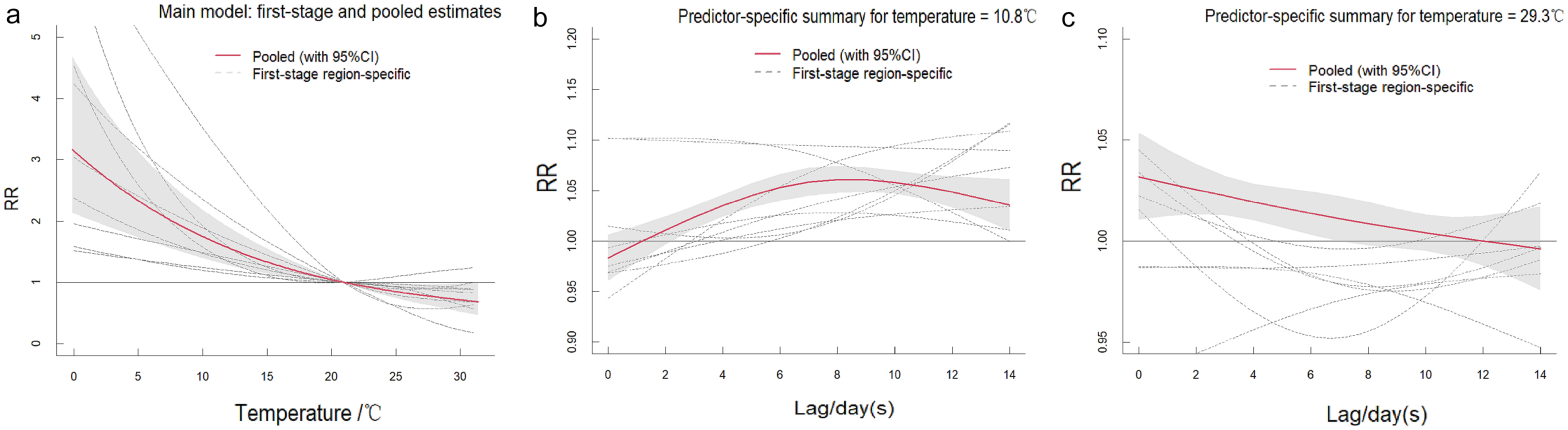
The pooled effects of temperature on ILI in Fujian, 2015–2023. The picture **(a)** shows the overall cumulative effects over lag 0–14 days in 9 cities, the two pictures describe **(b,c)** the pooled effects at predictor–specific (95th and 5th percentile of temperature). The dotted lines represent the different effects of 9 cities, the red line represents the pooled effect and the shaded area is the confidence interval (CI with 95%). The reference level was set to the median value of the corresponding variable.

The meta-analysis revealed high heterogeneity in both exposure and lag responses, with all multivariate Cochran’s *Q* tests indicating statistically significant differences (*p* < 0.05; Table 2). NO₂ significantly contributed to heterogeneity (reducing *I*^2^ to 86.6%), though residual heterogeneity persisted (*p* < 0.001). Pooled cumulative effects demonstrated a non-linear, inverted U-shaped association between NO₂ and ILI (Figure 6), with elevated risks (RR > 1) at concentrations of 19.1–66.3 μg/m^3^, peaking at 40.5 μg/m^3^ (RR = 1.426, 95% CI: 1.244–1.636) (Supplementary Table S7). Low NO₂ exposure (vs. 50th percentile) showed protective effects (cumulative RR = 0.697, 95% CI: 0.608–0.800), while high exposure increased risk [strongest at lag 1 day (RR = 1.032, 95% CI: 1.016–1.048); cumulative RR = 1.426 by lag 14] (Supplementary Table S8). Similarly, SO₂ exhibited a positive dose–response relationship with ILI, albeit with greater variability (Figure 7). Temperature effects followed an inverse pattern: pooled results identified peak risk at −0.1°C (RR = 3.165, 95% CI: 2.140–4.681) (Figure 8), with significant cumulative risks (RR > 1) below 20.9°C. Low temperatures (5th percentile) showed maximal lag 9-day effects (RR = 1.061, 95% CI: 1.049–1.073; cumulative RR = 1.863 by lag 14), whereas high temperatures (95th percentile) linearly reduced risk (lag 0 RR = 1.032, 95% CI: 1.011–1.054; cumulative RR = 0.731 at lag 14) (Supplementary Table S8).

**Table 2:** Cochran’s multivariate *Q* test and *I*^2^ statistic to assess heterogeneity in the meta-analytic models.

Meta–analytic model	*Q*	df	*P*	*I*^2^ (%)
Overall cumulative response				
P50_NO₂	178.90	24	<0.001	86.6
P50_SO₂	656.34	24	<0.001	96.3
P50_Tem	346.11	24	<0.001	93.1
Lag response				
P5_Tem	320.75	24	<0.001	92.5
P95_Tem	245.08	24	<0.001	90.2
P5_NO₂	295.47	24	<0.001	91.9
P95_NO₂	147.13	24	<0.001	83.7
P5_SO₂	302.50	24	<0.001	92.1
P95_SO₂	458.82	24	<0.001	94.8

*Q*–statistics/*P*/*I*^2^, indicators for quantifying the heterogeneity of environmental factors in various cities; P5/P95 value; The P5/P95 value is the 5th/95th Percentile of environmental values during the study period, which can represent can represent extremely low/high exposure environmental conditions.

### 3.5 Sensitivity analysis results

The parameter estimates from the meta-analysis (Supplementary Table S9) and the RR values corresponding to the single-lag associations of the three environmental factors (Supplementary Table S10) remained consistent across all sensitivity analyses. Notably, the exposure-response curves exhibited minimal variation (Supplementary Figures S11–S13), confirming both the stability of our model and the appropriateness of the selected variables.

## 4 Discussion

Influenza-like illness represents a significant global public health challenge, with transmission dynamics strongly influenced by environmental factors (14). This study systematically examines the associations between environmental exposures and ILI incidence across nine prefecture-level cities in Fujian Province, China. As a southeastern coastal region, Fujian exhibits marked variability in climate conditions and pollution levels, providing an ideal setting to investigate environmental drivers of ILI. The inclusion of all prefecture-level cities ensures a comprehensive assessment of provincial ILI patterns while capturing region-specific environmental heterogeneity.

Our analysis of ILI cases across nine Fujian Province cities revealed distinct bimodal seasonality, with primary peaks occurring during winter months (December–February) and secondary peaks in early summer (May–June), consistent with national surveillance data (25). This pattern likely reflects enhanced viral survival and transmission under winter’s lower temperatures and higher humidity (5).

Consistent with existing literature demonstrating the influence of meteorological factors and air pollutants on ILI prevalence (1, 11, 26, 27). we observed elevated ILI risk at moderate-to-high NO₂ concentrations (30–60 μg/m^3^) with short lag periods (0–6 days), suggesting acute effects of NO₂ exposure. This risk pattern attenuated substantially after >10 days of exposure, indicating time-dependent effects.

Our multi-city analysis demonstrated an inverted U-shaped concentration-response relationship between NO₂ exposure and ILI incidence. Cumulative exposure assessment revealed that short-term, low-concentration NO₂ exposure (<30 μg/m^3^) was associated with reduced ILI risk, whereas prolonged high-concentration exposure (>60 μg/m^3^) significantly increased risk. These findings corroborate previous reports by Sun et al. on the association between air pollutants (PM2.5, NO₂, PM10, and SO₂) and influenza risk (28), as well as observations from Ningbo regarding NO₂ and O₃ exposure effects (29). Mechanistically, NO₂ acts as a potent respiratory irritant that induces airway inflammation and oxidative stress, potentially compromising immune defenses and increasing susceptibility to respiratory infections (30, 31).

The influence of temperature on ILI risk exhibits regional variability, yet an overall trend emerges: ILI risk is elevated at low-to-moderate temperatures and longer lag times but diminishes at higher temperatures, with the most pronounced effects occurring during short lag periods. Analysis of temperature–ILI associations across nine cities reveals that rising temperatures correlate with reduced ILI risk, whereas prolonged exposure to cold temperatures (below the 5th percentile) increases susceptibility. These findings align with Chow et al.’s study in Mississippi, which reported that low temperatures (lag 0–4 weeks) heightened ILI risk, while high temperatures had negligible effects on transmission (1). Similarly, Yang et al. demonstrated that cold weather not only elevates ILI risk but also accounts for a substantial proportion of the disease burden (7). Similar findings have been validated in several cities across China (32–34). Various hypotheses have been proposed to explain the relationship between low temperatures and influenza. Low temperatures facilitate the transmission of influenza in multiple ways, such as by affecting virus survival and spread, influencing host susceptibility, and altering human behavior and the environment (35, 36). When temperatures drop, the stability of influenza virus particles increases, and changes in the physical properties of the viral envelope promote the survival and spread of the virus. While most studies indicate that low temperatures significantly enhance the risk of ILI, some suggest that high temperatures can also be a risk factor for ILI. For instance, Tamerius et al. (37) found that influenza outbreaks in tropical and subtropical regions occur during hot and humid periods. Additionally, Grigorieva and Lukyanets (38) discovered that the combined effect of high temperatures and air pollutants promoted the occurrence of respiratory diseases like influenza. These disparities highlight the potential influence of climatic zones, socioeconomic factors, and lifestyle habits on ILI transmission dynamics.

The findings of this study hold significant academic and public health implications. Against the backdrop of rapid economic development and ongoing urbanization in China, air pollution has emerged as a major environmental risk factor impacting population health (39). This study innovatively integrated data from all prefecture-level cities in Fujian Province, overcoming the limitations of previous single-city studies with restricted sample sizes, and is the first to systematically verify the non-linear and delayed effects of environmental factors on ILI at a provincial scale. The results indicate that air pollutants such as NO₂ and SO₂ can significantly increase the risk of ILI incidence. This finding provides critical multi-center empirical evidence for research on the health effects of air pollution in China, particularly filling a research gap in subtropical coastal regions. Furthermore, this study confirms that low temperature exposure is an important risk factor for ILI. In the context of increasing extreme weather events due to climate change, this result carries substantial warning implications: extreme cold events may trigger clustered ILI outbreaks, thereby intensifying the burden on healthcare systems. Based on these findings, we recommend: (a) continuing to strengthen air pollution control, with particular focus on the management of NO₂ and SO₂ emissions; (b) enhancing preemptive deployment and reinforcement of ILI prevention and intervention measures during cold wave alerts; and (c) formulating differentiated prevention and control strategies for respiratory infectious diseases tailored to the characteristics of different climate zones. This study not only provides new scientific evidence for environmental epidemiology but also offers a valuable reference for public health authorities in developing targeted intervention strategies. Future research should further explore the synergistic health effects of air pollution and climate change to better address the health challenges posed by complex environmental changes.

In summary, this study reinforces the significant role of air pollutants and meteorological factors in ILI transmission, underscoring the need for enhanced air quality control and weather monitoring during influenza seasons. However, several limitations must be acknowledged. First, the air pollutant data were derived from outdoor monitoring stations, which may not precisely reflect individual exposure levels, potentially introducing measurement bias. Second, since ILI cases were identified based on sentinel hospital reports, mild or subclinical infections may have been excluded, leading to an underestimation of the true disease burden. Additionally, due to data constraints, other potentially influential meteorological variables—such as solar radiation and absolute humidity—were not incorporated into the analysis. To address these gaps, future studies should integrate more precise individual-level exposure assessments to better characterize the relationship between air pollution and ILI. Furthermore, combining epidemiological data with laboratory-based investigations could help elucidate the underlying biological mechanisms driving these associations.

## 5 Conclusion

This multi-city study in Fujian Province revealed ILI exhibits winter/early summer peaks, with short-term NO₂ exposure and prolonged low-temperature exposure significantly increasing risk. The extended cold-weather lag effect highlights the need for integrated prevention strategies combining air quality and meteorological monitoring during influenza seasons.

## Data Availability

The original contributions presented in the study are included in the article/Supplementary material, further inquiries can be directed to the corresponding author.

## Footnotes

^1^ https://www.aqistudy.cn/historydata/

^2^ http://data.cma.cn/
