# Supplementary material for: Impact of multiple environmental factors on influenza–like illness in Fujian Province, China, 2015–2023: a multicity study
Source: Front Public Health. 2025 Sep 4;13:1656880. doi: 10.3389/fpubh.2025.1656880 (PMC12443796; doi:10.3389/fpubh.2025.1656880)
Supplement: Supplementary file 1 [file Data_Sheet_1.ZIP › Supplementary Attachment/Supplementary Figure Attachment.docx]

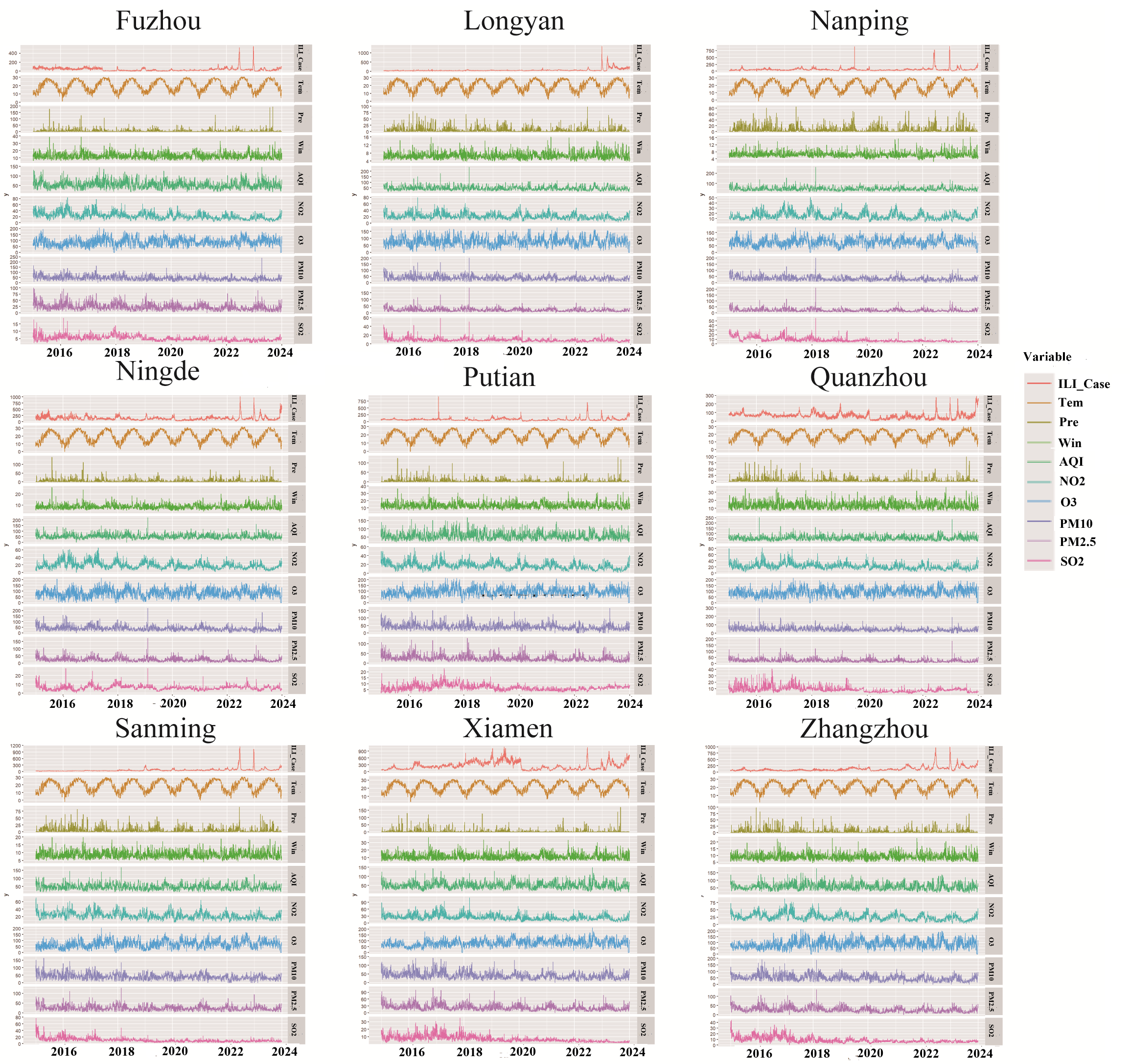


**Figure S1. Time series plots of daily ILI cases, meteorological factors, and air pollution for 9 cities in Fujian Province from 2015 to 2023.**


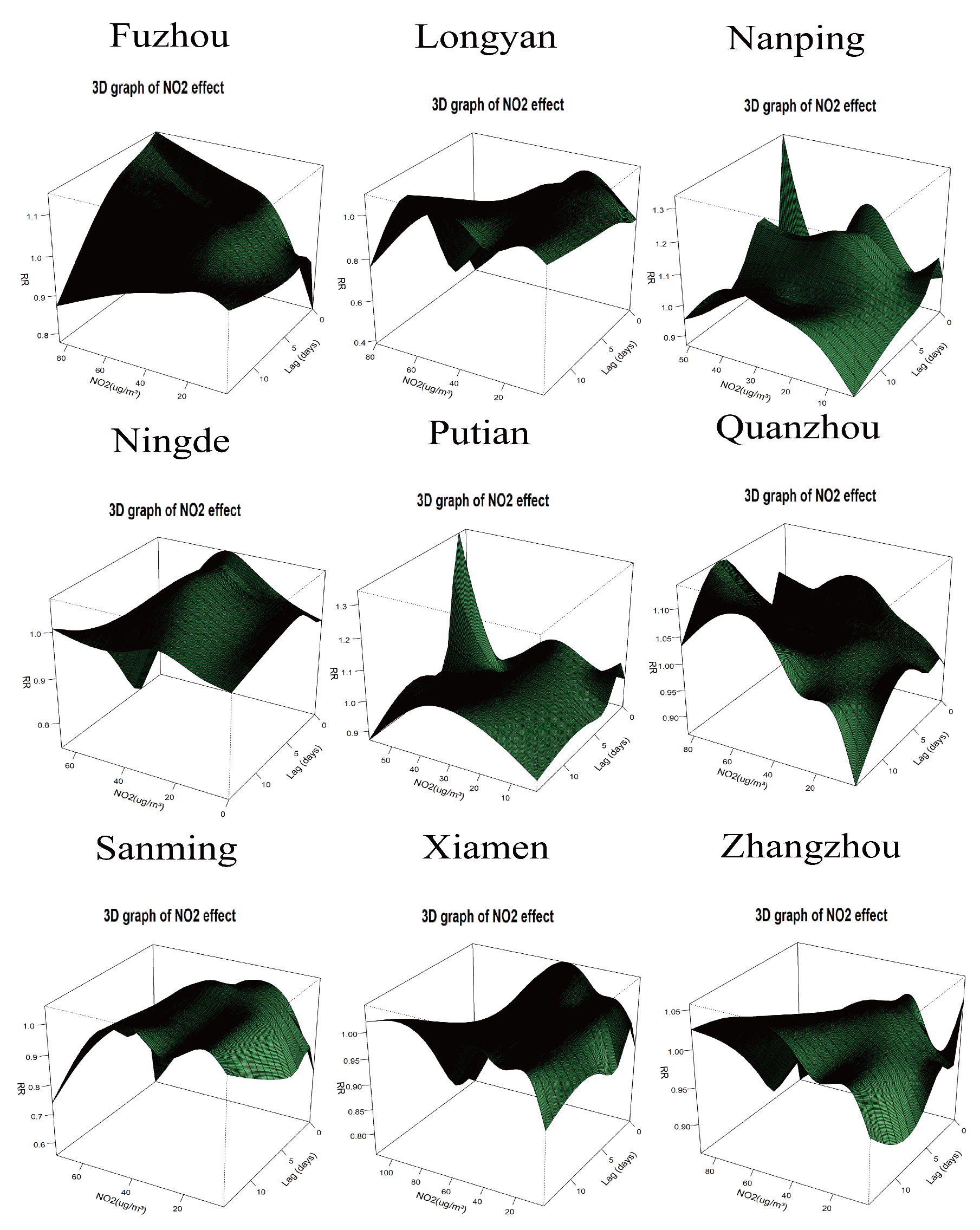


Figure S2. Three-dimensional graph of the relative risks of NO₂ on ILI cases in 9 cities in Fujian Province from 2015 to 2023. The reference level was set to the median value of NO₂. The Z-axis represents the lag period from 0 to 14 days. The X-axis represents the range of observations for NO₂. RR stands for relative risk.


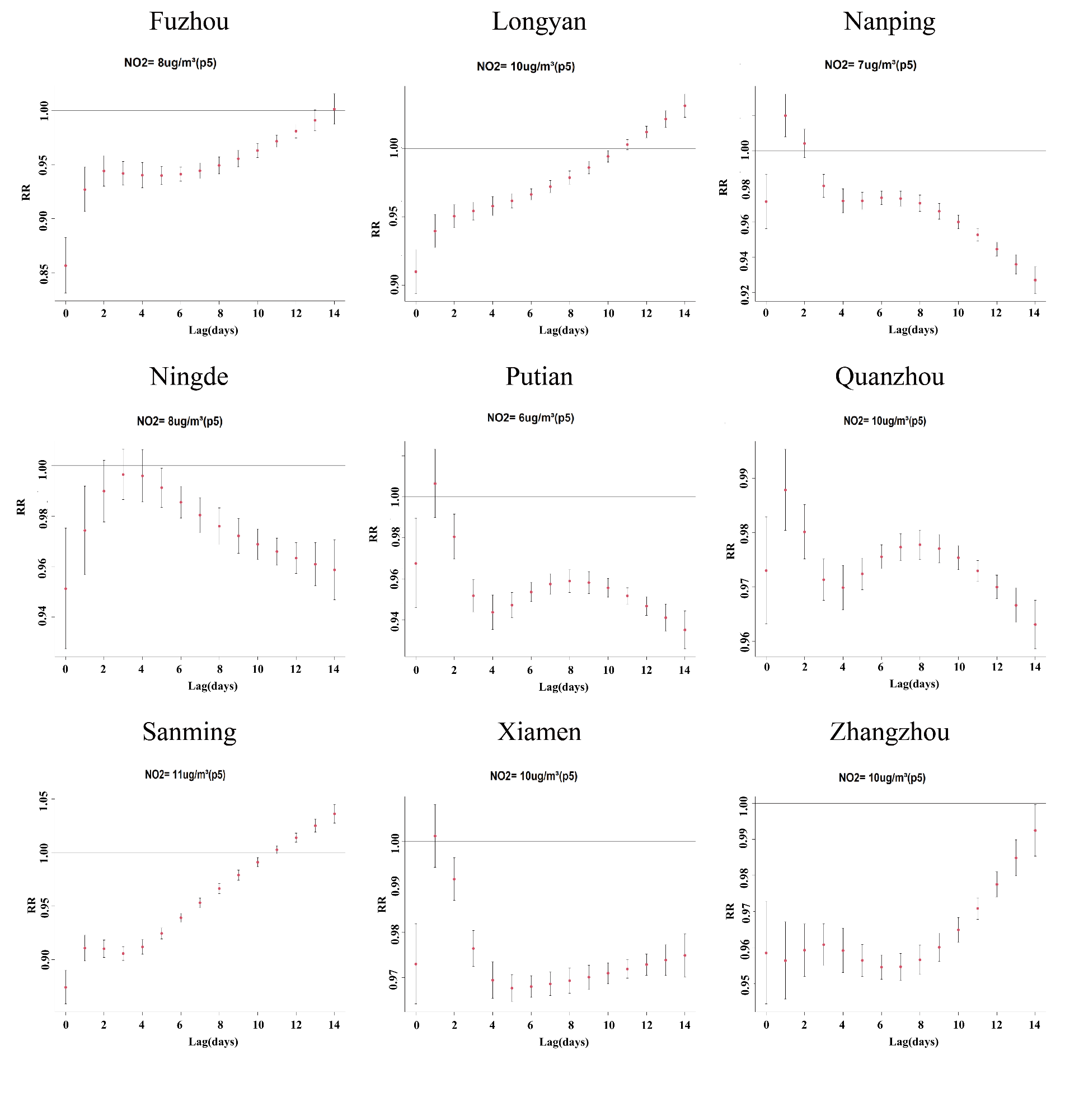


Figure S3. Scatter plots of exposure-response relationships between ILI and P5_NO₂ in the Multi-environmental variable model in 9 cities in Fujian Province from 2015 to 2023. The reference level was set to the median value of the corresponding variable. The X-axis represents the lag period from 0 to 14 days. RR stands for relative risk. The P5_NO₂ value is the 5th Percentile of NO₂ during the study period, which can represent can represent extremely low NO₂ exposure conditions.


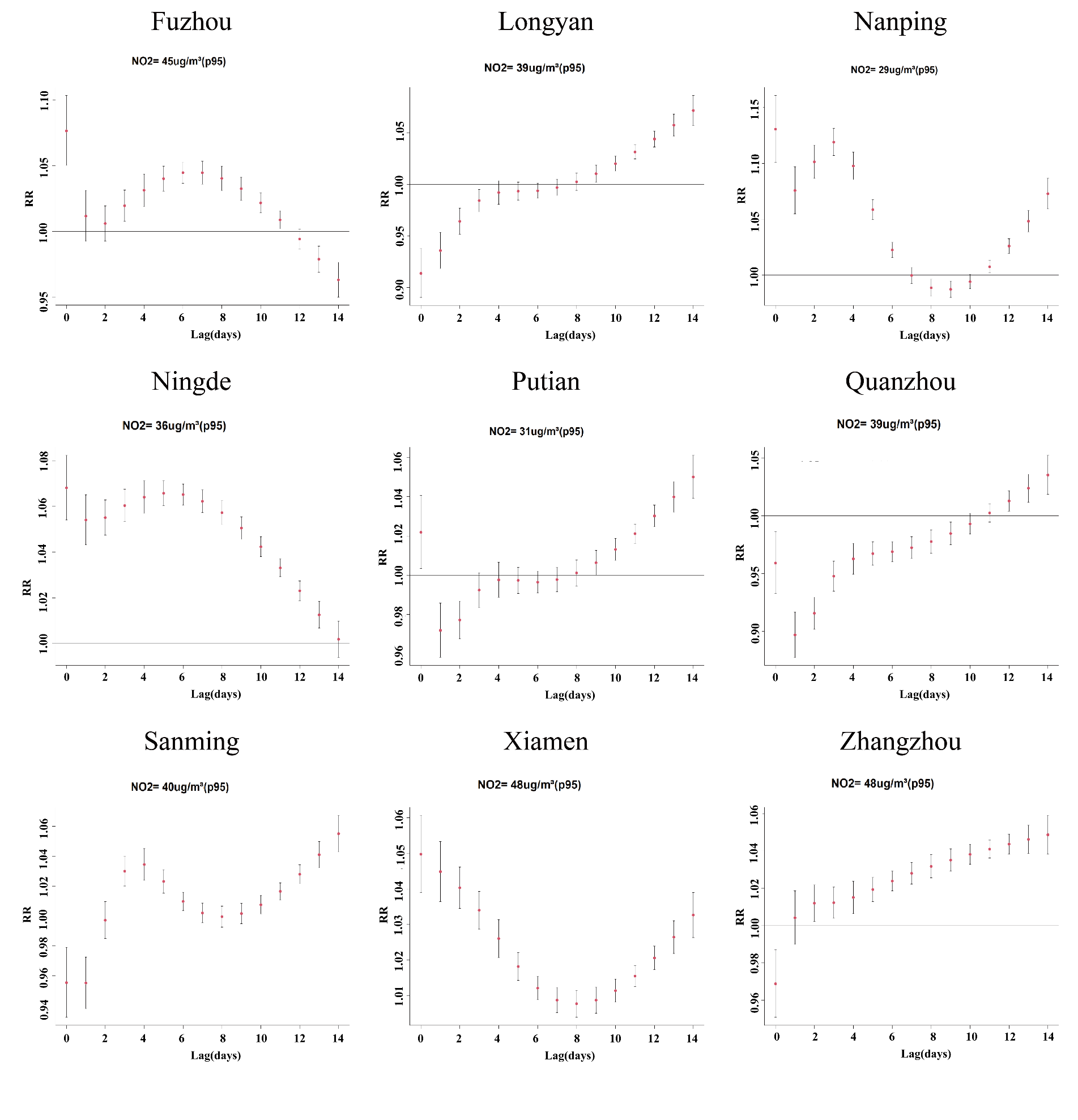


Figure S4. Scatter plots of exposure-response relationships between ILI and P95_NO₂ in the Multi-environmental variable model in 9 cities in Fujian Province from 2015 to 2023. The reference level was set to the median value of the corresponding variable. The X-axis represents the lag period from 0 to 14 days. RR stands for relative risk. The P95_NO₂ value is the 95th Percentile of NO₂ during the study period, which can represent can represent extremely high NO₂ exposure conditions.


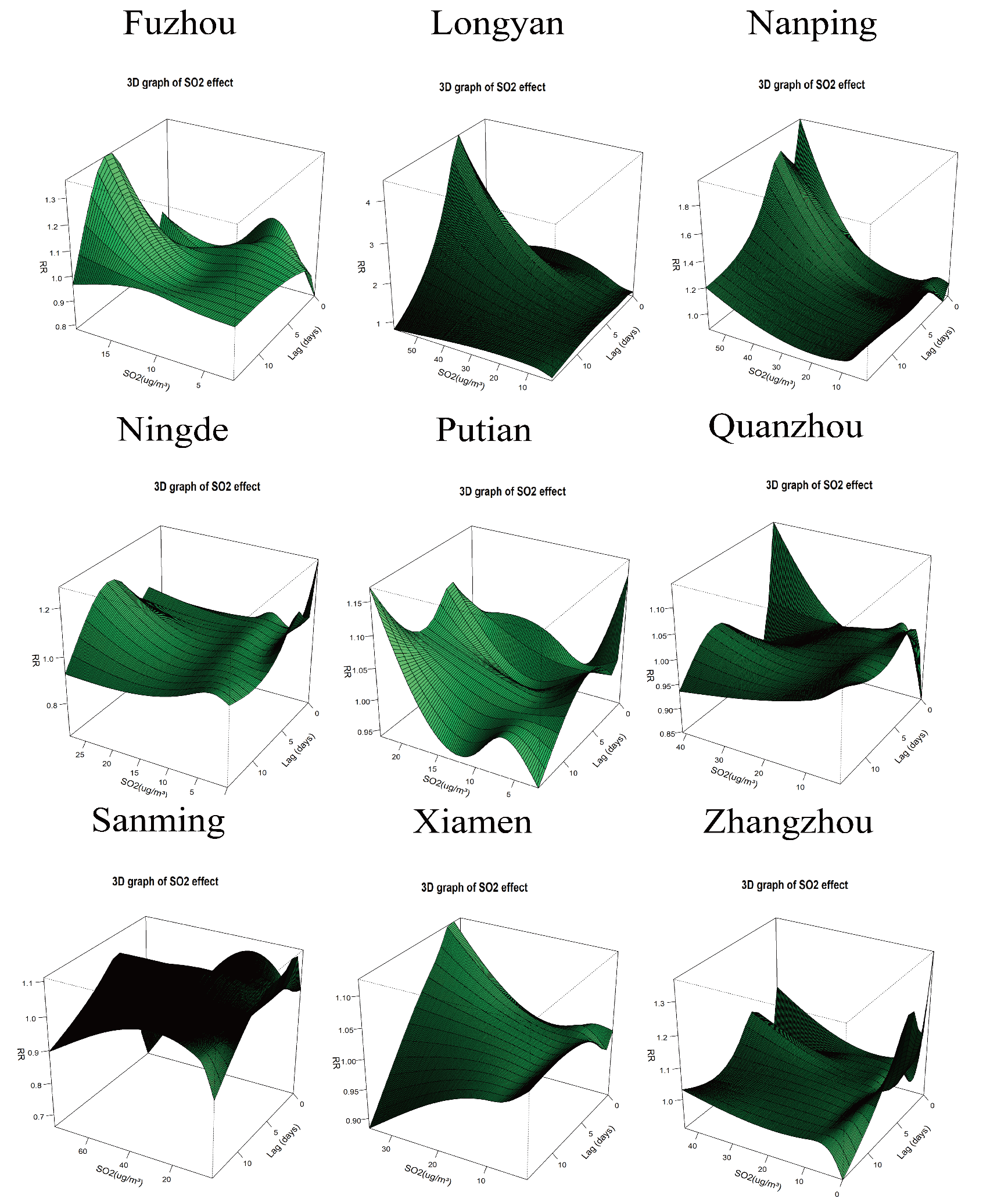


Figure S5. Three-dimensional graph of the relative risks of SO₂ on ILI cases in 9 cities in Fujian Province from 2015 to 2023. The reference level was set to the median value of SO₂. The Z-axis represents the lag period from 0 to 14 days. The X-axis represents the range of observations for SO₂. RR stands for relative risk.


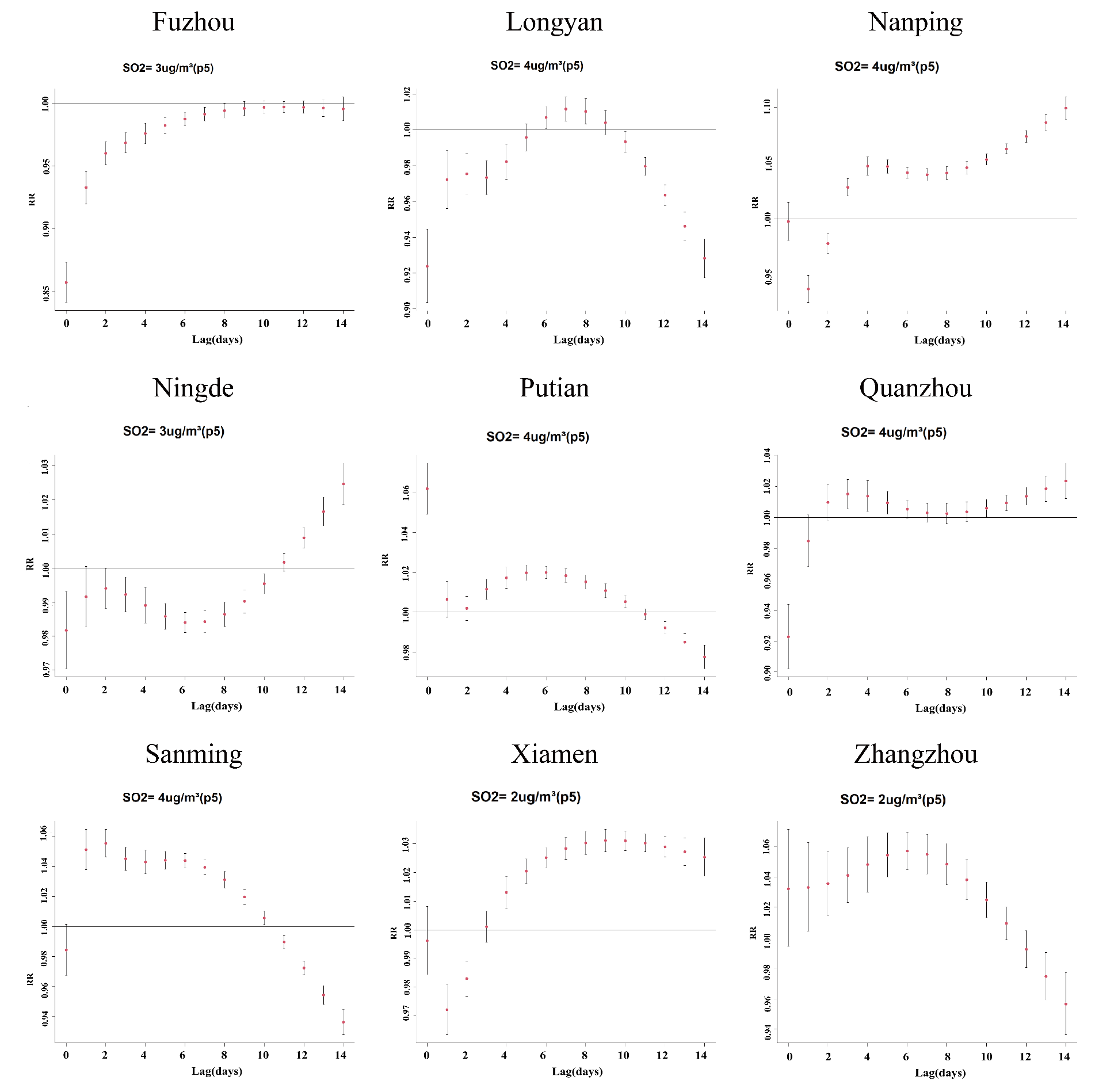


Figure S6. Scatter plots of exposure-response relationships between ILI and P5_SO₂ in the Multi-environmental variable model in 9 cities in Fujian Province from 2015 to 2023. The reference level was set to the median value of the corresponding variable. The X-axis represents the lag period from 0 to 14 days. RR stands for relative risk. The P5_SO₂ value is the 5th Percentile of SO₂ during the study period, which can represent can represent extremely low SO₂ exposure conditions.


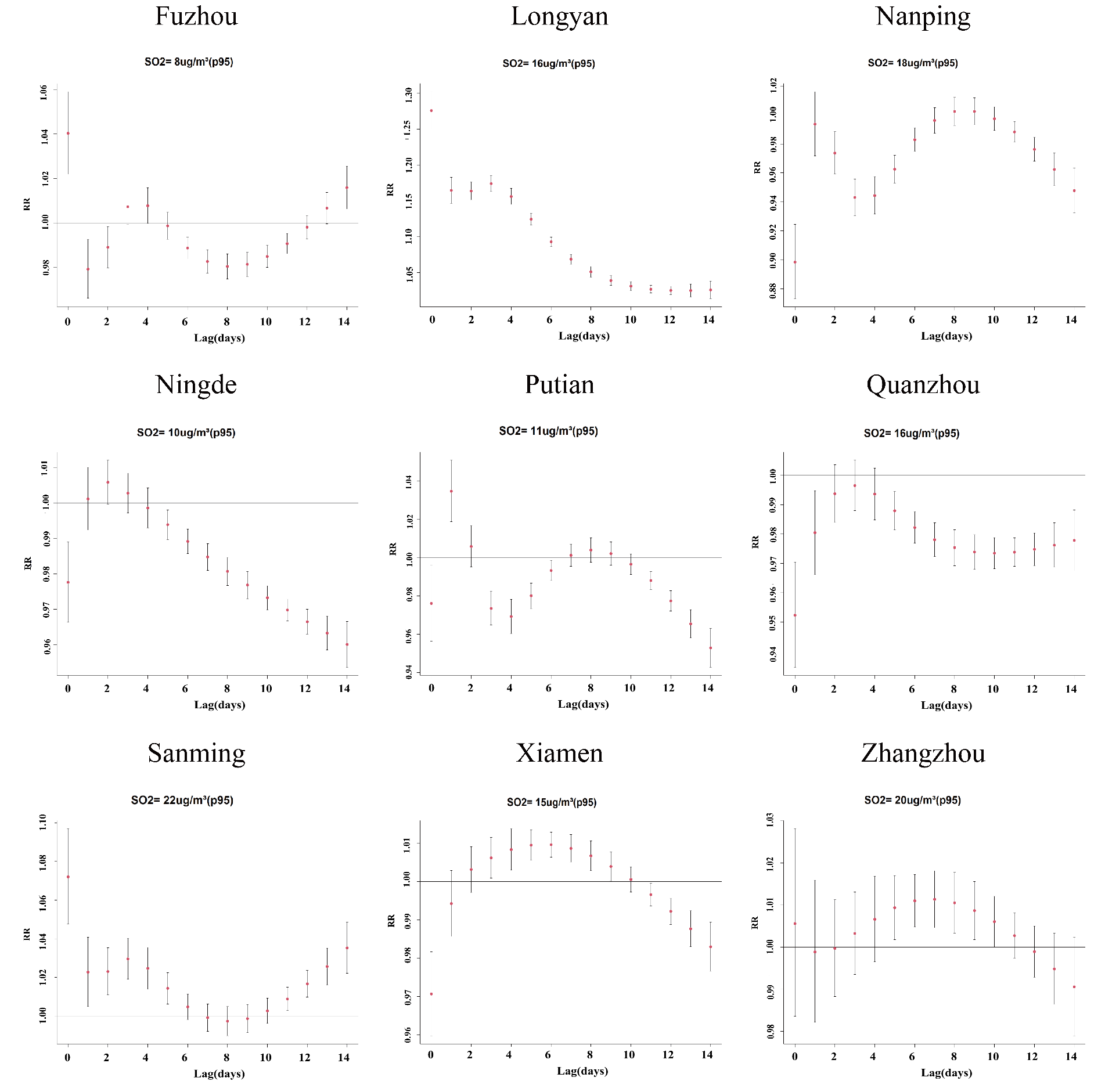


Figure S7. Scatter plots of exposure-response relationships between ILI and P95_SO₂ in the Multi-environmental variable model in 9 cities in Fujian Province from 2015 to 2023. The reference level was set to the median value of the corresponding variable. The X-axis represents the lag period from 0 to 14 days. RR stands for relative risk. The P95_SO₂ value is the 95th Percentile of SO₂ during the study period, which can represent can represent extremely high SO₂ exposure conditions.


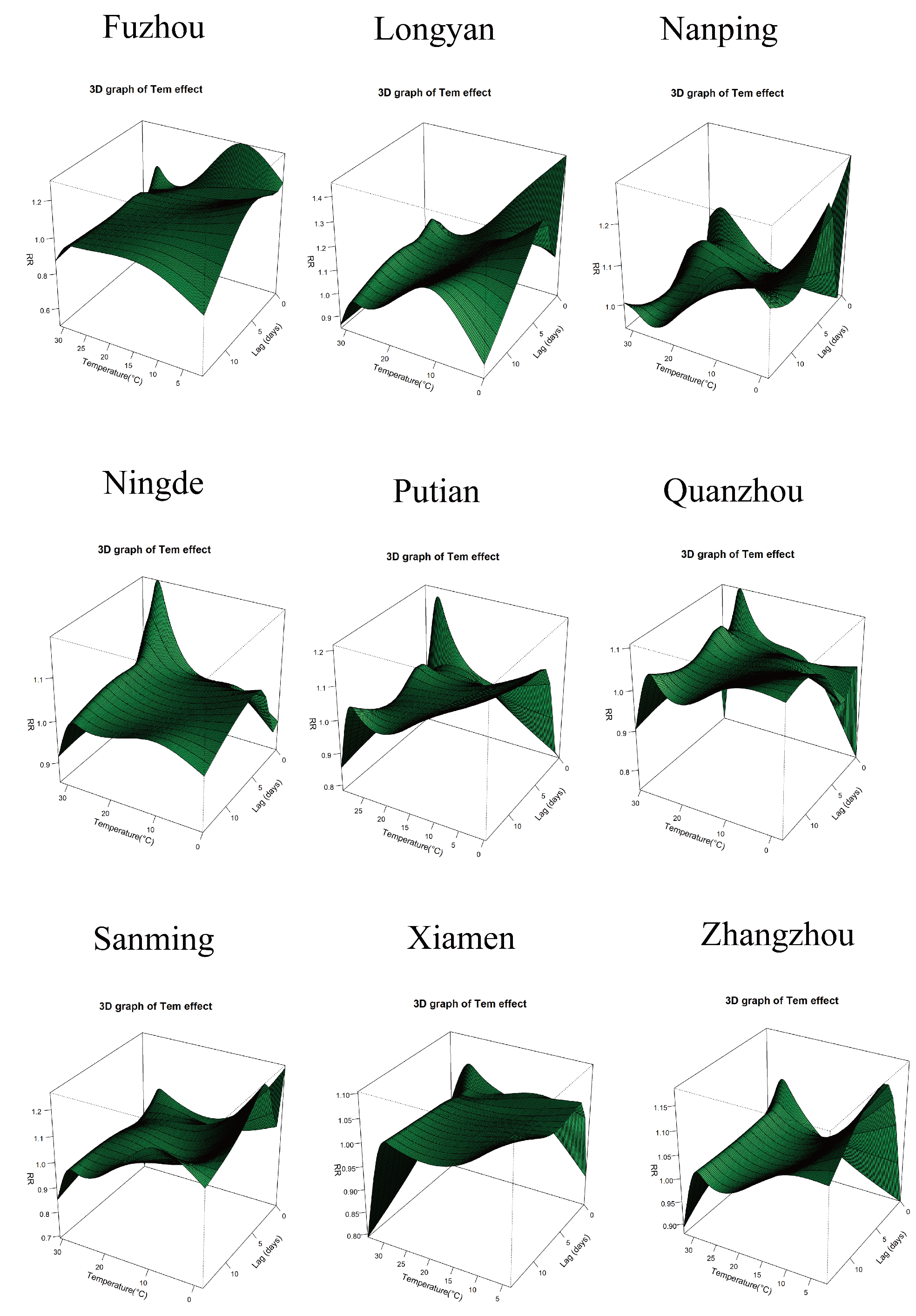


Figure S8. Three-dimensional graph of the relative risks of Temperature on ILI cases in 9 cities in Fujian Province from 2015 to 2023. The reference level was set to the median value of Temperature. The Z-axis represents the lag period from 0 to 14 days. The X-axis represents the range of observations for Temperature. RR stands for relative risk.


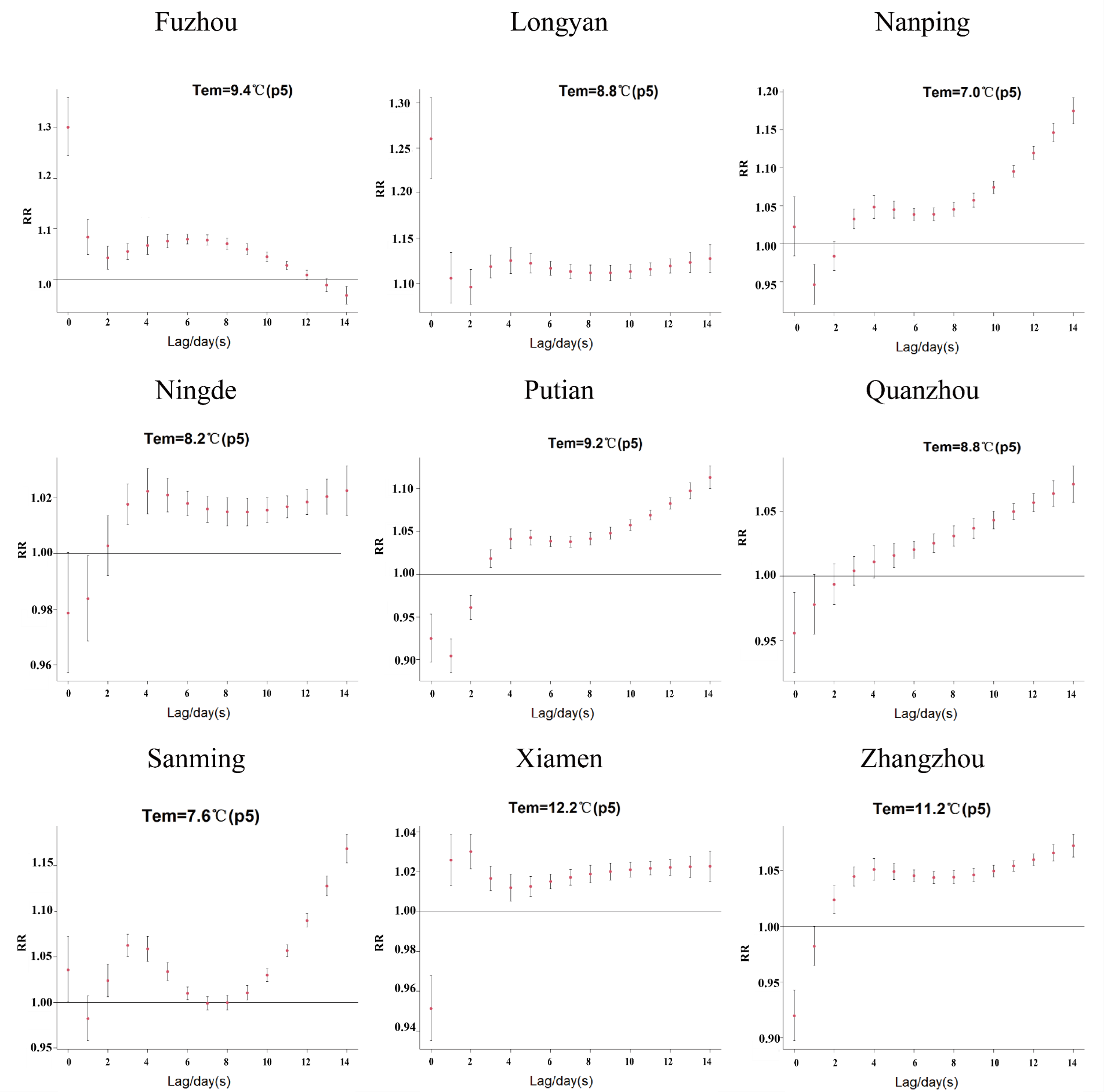


Figure S9. Scatter plots of exposure-response relationships between ILI and P5_Tem in the Multi-environmental variable model in 9 cities in Fujian Province from 2015 to 2023. The reference level was set to the median value of the corresponding variable. The X-axis represents the lag period from 0 to 14 days. RR stands for relative risk. The P5_Tem value is the 5th Percentile of temperature during the study period, which can represent can represent extremely low temperature exposure conditions.


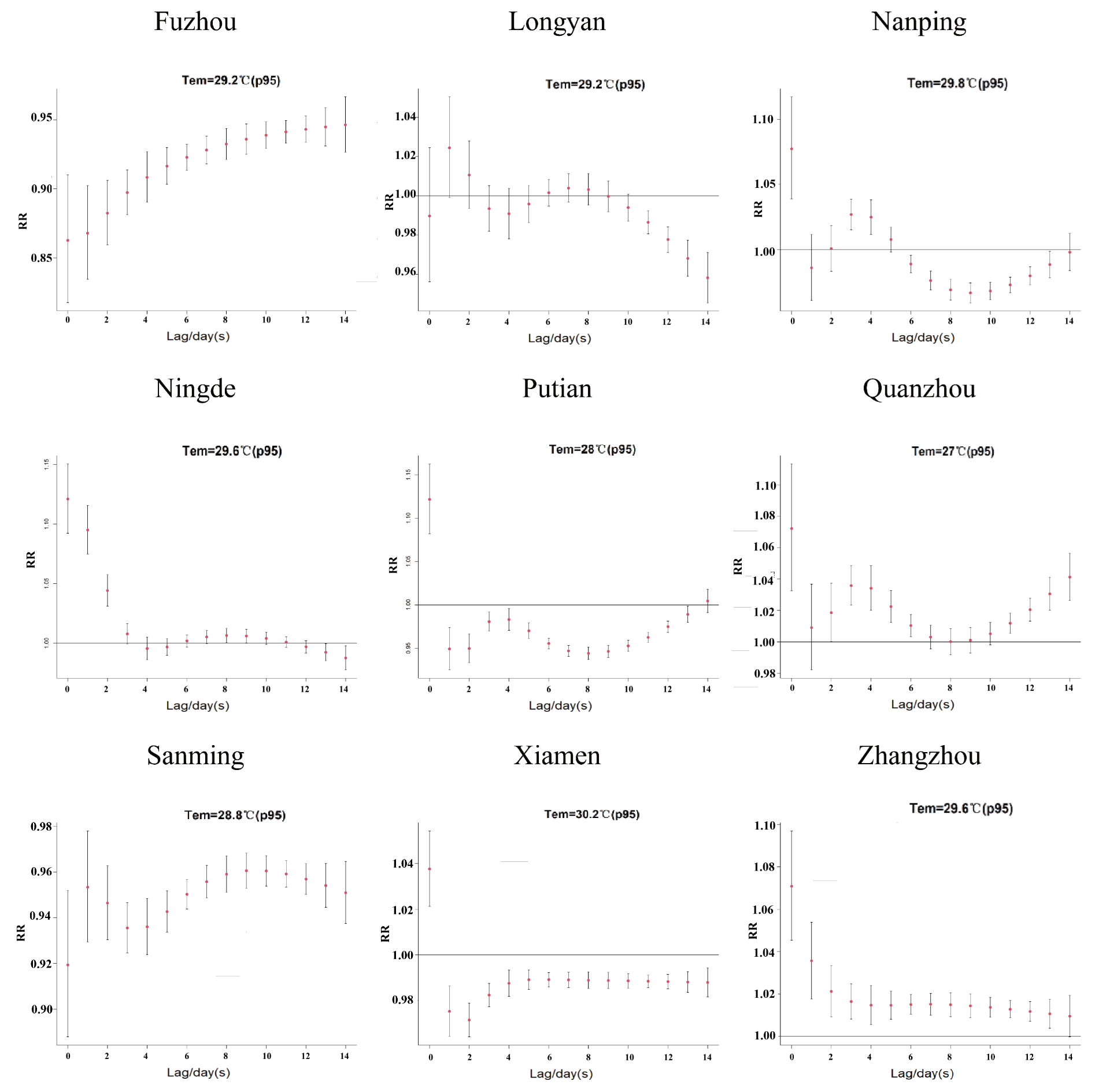


Figure S10. Scatter plots of exposure-response relationships between ILI and P95_Tem in the Multi-environmental variable model in 9 cities in Fujian Province from 2015 to 2023. The reference level was set to the median value of the corresponding variable. The X-axis represents the lag period from 0 to 14 days. RR stands for relative risk. The P95_Tem value is the 95th Percentile of temperature during the study period, which can represent can represent extremely high temperature exposure conditions.


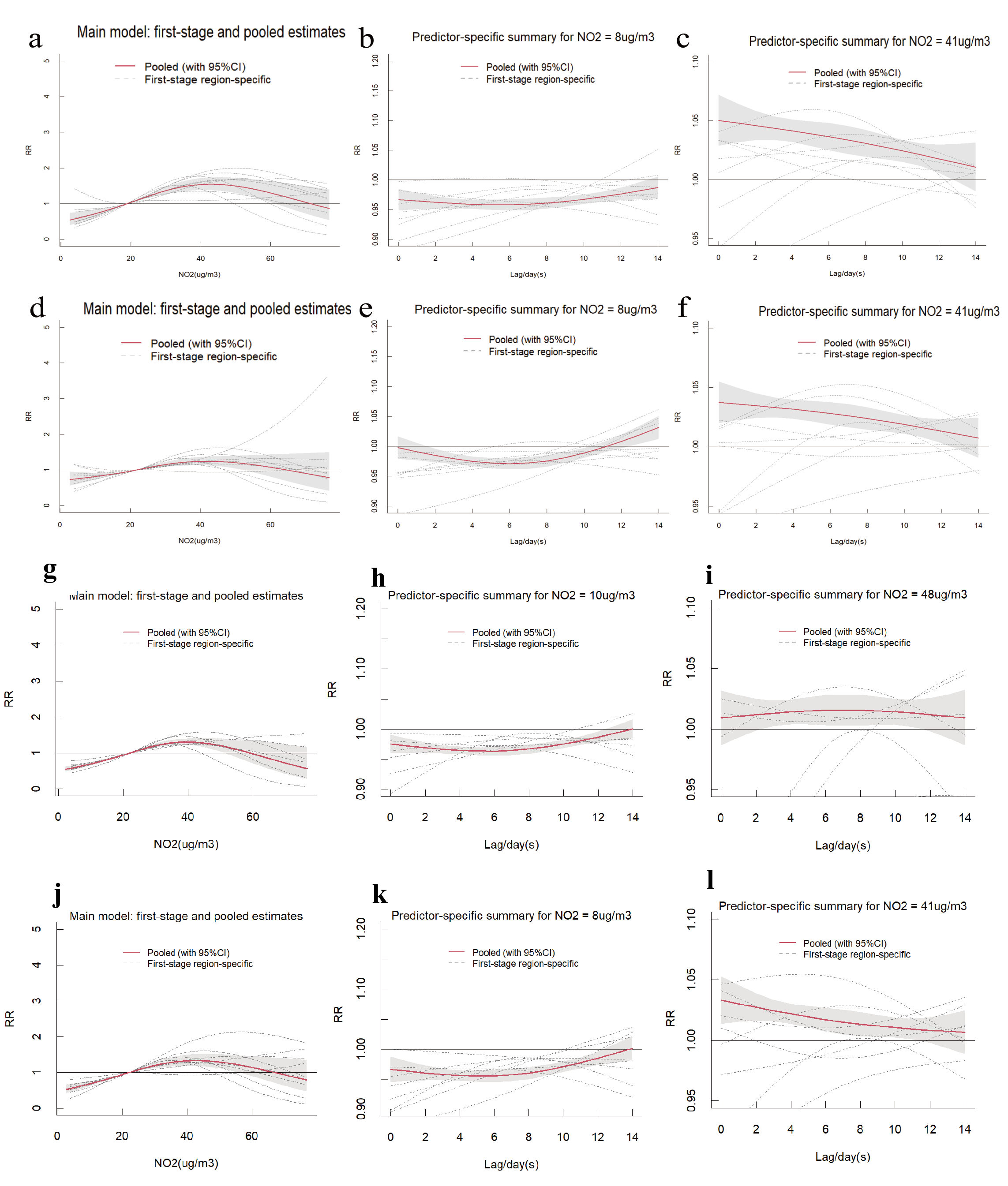


Figure S11. The sensitive analysis of pooled effects of NO₂ on ILI in Fujian, 2015–2023. The picture (a) shows the overall cumulative effects over lag 0–14 days in 9 cities on df value of time at 6, the two pictures describe (b, c) the pooled effects at predictor-specific (95th and 5th percentile of NO₂) on df value of time at 6. The picture (d) shows the overall cumulative effects over lag 0–14 days in 9 cities on df value of time at 8, the two pictures describe (e, f) the pooled effects at predictor-specific (95th and 5th percentile of NO₂) on df value of time at 8. Pictures (g, h, i) show the overall cumulative effect, the 95th percentile, and the 5th percentile of NO₂ summary effect of the 7 cities within a lag of 0–14 days after excluding cities with extreme population structures. Pictures (j, k, l) show the overall cumulative effect, the 95th percentile, and the 5th percentile of NO₂ summary effect of the 9 cities within a lag of 0–14 days after adding covariate modeling.The dotted lines represent the different effects of 9 cities, the red line represents the pooled effect and the shaded area is the confidence interval (CI with 95%). The reference level was set to the median value of the corresponding variable.


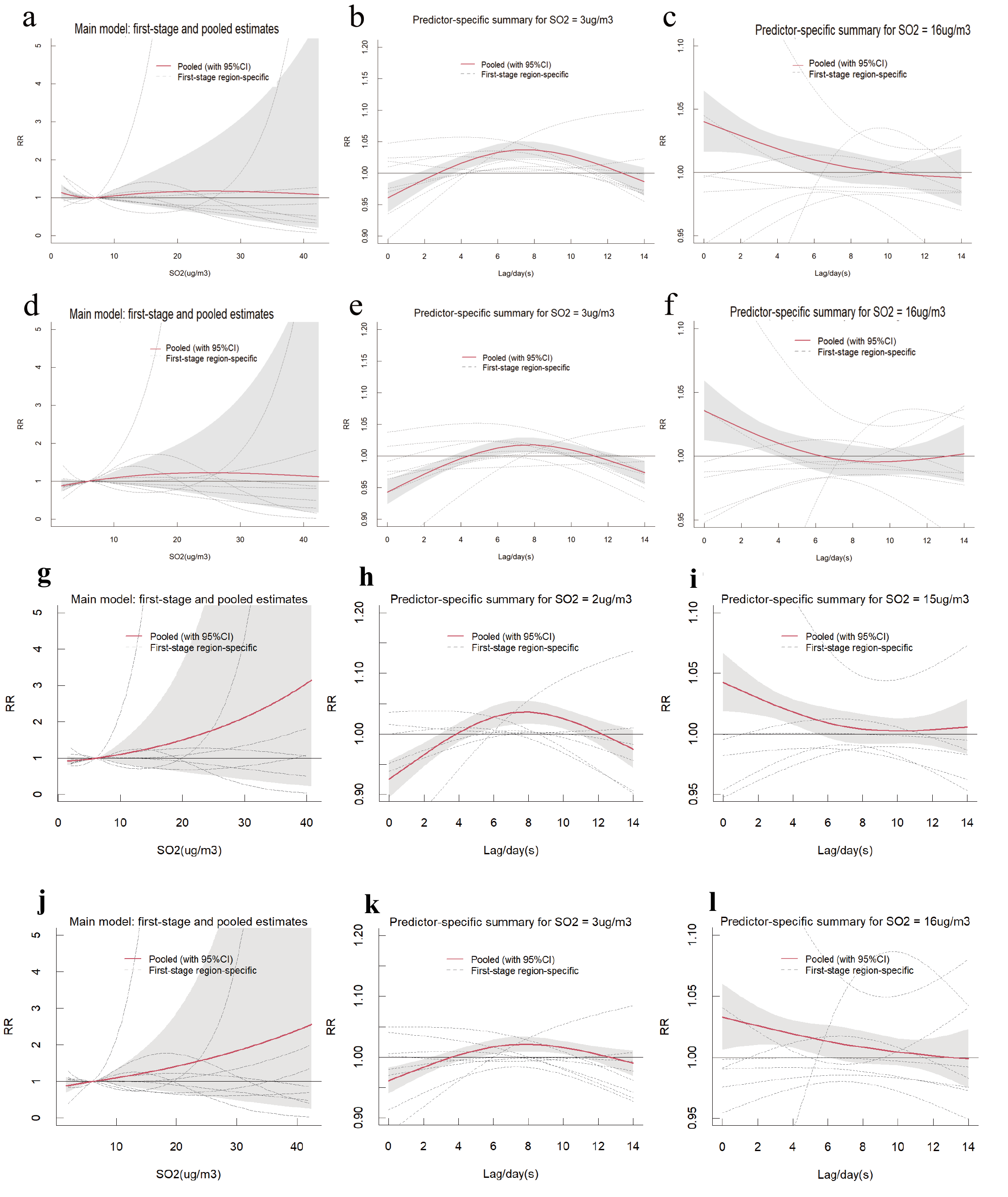


Figure S12. The sensitive analysis of pooled effects of SO₂ on ILI in Fujian, 2015–2023. The picture (a) shows the overall cumulative effects over lag 0–14 days in 9 cities on df value of time at 6, the two pictures describe (b, c) the pooled effects at predictor-specific (95th and 5th percentile of SO₂) on df value of time at 6. The picture (d) shows the overall cumulative effects over lag 0–14 days in 9 cities on df value of time at 8, the two pictures describe (e, f) the pooled effects at predictor-specific (95th and 5th percentile of SO₂) on df value of time at 8. Pictures (g, h, i) show the overall cumulative effect, the 95th percentile, and the 5th percentile of SO₂ summary effect of the 7 cities within a lag of 0–14 days after excluding cities with extreme population structures. Pictures (j, k, l) show the overall cumulative effect, the 95th percentile, and the 5th percentile of SO₂ summary effect of the 9 cities within a lag of 0–14 days after adding covariate modeling.The dotted lines represent the different effects of 9 cities, the red line represents the pooled effect and the shaded area is the confidence interval (CI with 95%). The reference level was set to the median value of the corresponding variable.


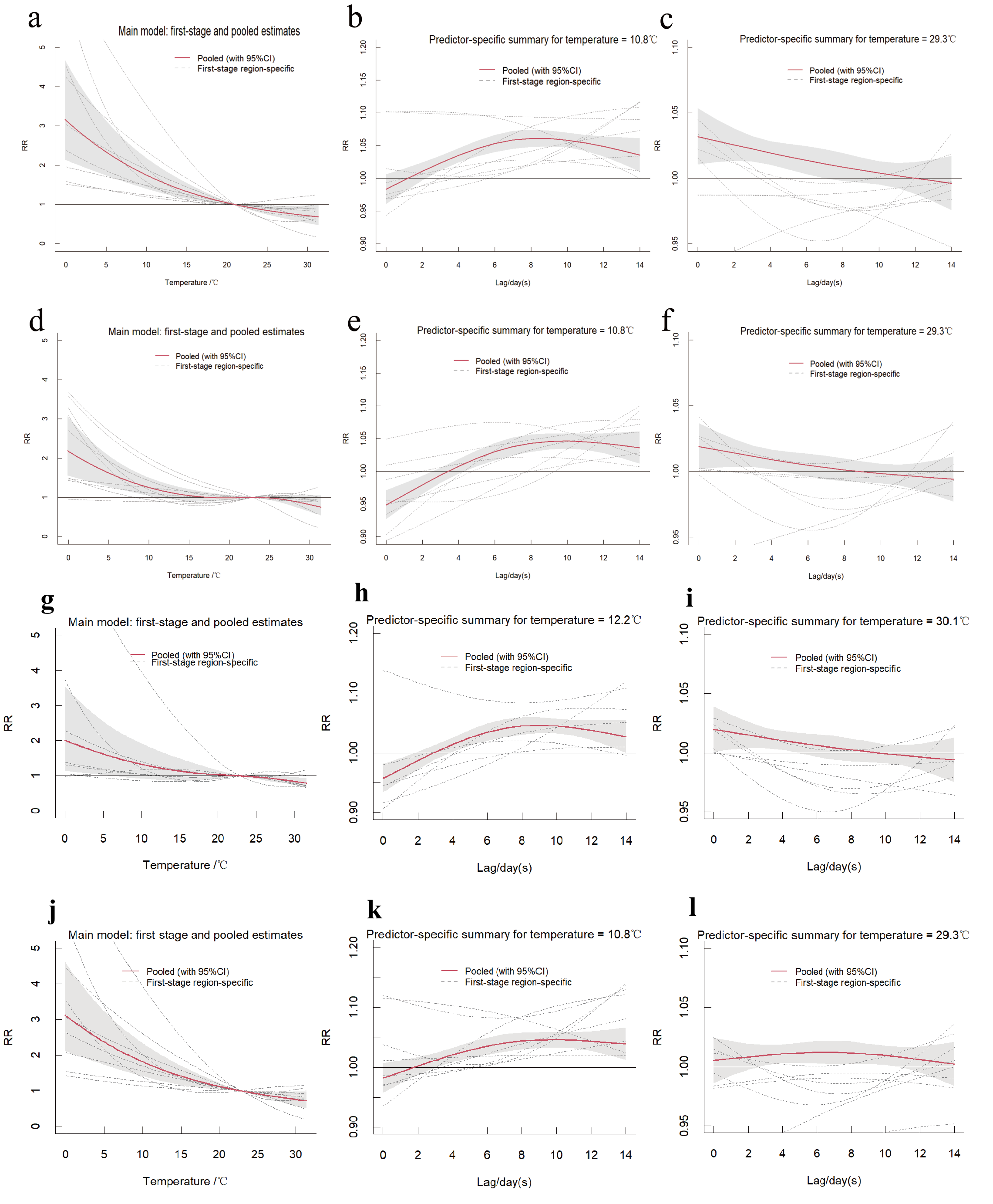


Figure S13. The sensitive analysis of pooled effects of temperature on ILI in Fujian, 2015–2023. The picture (a) shows the overall cumulative effects over lag 0–14 days in 9 cities on df value of time at 6, the two pictures describe (b, c) the pooled effects at predictor-specific (95th and 5th percentile of temperature) on df value of time at 6. The picture (d) shows the overall cumulative effects over lag 0–14 days in 9 cities on df value of time at 8, the two pictures describe (e, f) the pooled effects at predictor-specific (95th and 5th percentile of temperature) on df value of time at 8. Pictures (g, h, i) show the overall cumulative effect, the 95th percentile, and the 5th percentile of temperature summary effect of the 7 cities within a lag of 0–14 days after excluding cities with extreme population structures. Pictures (j, k, l) show the overall cumulative effect, the 95th percentile, and the 5th percentile of temperature summary effect of the 9 cities within a lag of 0–14 days after adding covariate modeling.The dotted lines represent the different effects of 9 cities, the red line represents the pooled effect and the shaded area is the confidence interval (CI with 95%). The reference level was set to the median value of the corresponding variable.
